# CHANGE IN DEMENTIA FAMILY CAREGIVERS’ WILLINGNESS TO PAY FOR A NONPHARMACOLOGIC INTERVENTION

**DOI:** 10.1093/geroni/igz038.2030

**Published:** 2019-11-08

**Authors:** Eric Jutkowitz, Daniel Scerpella, Katherine Prioli, Katherine Marx, Laura N Gitlin, Laura Pizzi, Jonah Popp

**Affiliations:** 1 Brown University, Providence, Rhode Island, United States; 2 Johns Hopkins University, Baltimore, Maryland, United States; 3 Rutgers University, Piscataway, Maryland, United States; 4 Drexel University, Philadelphia, Pennsylvania, United States

## Abstract

Family caregivers provide a majority of care for persons with dementia (PwD); however, little is known about caregiver’s willingness to pay (WTP) for an intervention to help them manage dementia symptoms. To fill this gap, caregiver/PwD dyads (n=223) were recruited to participate in a randomized trial evaluating tailored activities to minimize behavioral symptoms and functional decline. At baseline and 6-months caregivers were asked their WTP per session for the 8-session 3-month program compared to caregiver education/support only. At baseline, treatment caregivers were WTP $26.20, which was $11.50 (95%CI:-$12.70, -$10.3) less per session compared to control group caregivers WTP $37.30. At 6-months, treatment caregivers were WTP $22.90 and control caregivers $27.30. From baseline to 6-months, a change in WTP was $7.00 (95%CI:$5.80, $8.30) greater than the change in WTP for control group caregivers. Caregivers WTP slightly decreases over time in both groups but decrease is less for TAP following program participation.

